# P-1140. Predictive Score for “True” Coagulase-negative Staphylococcus Bloodstream Infections

**DOI:** 10.1093/ofid/ofaf695.1334

**Published:** 2026-01-11

**Authors:** Beatriz Arns, Flávia R Brust, Daniel Sganzerla, Mateus Swarovsky Helfer, Vlademir V Cantarelli, Alexandre Zavascki

**Affiliations:** Hospital Moinhos de Vento, Porto Alegre, Rio Grande do Sul, Brazil; Health Security Partners, ´Porto Alegre, Rio Grande do Sul, Brazil; Inova Medical Research, Porto Alegre, Rio Grande do Sul, Brazil; Hospital de Clinicas de Porto Alegre, Porto Alegre, Rio Grande do Sul, Brazil; Federal University of Health Sciences of Porto Alegre, Cachoeirinha, Rio Grande do Sul, Brazil; Hospital de Clínicas de Porto Alegre, Porto Alegre, Rio Grande do Sul, Brazil

## Abstract

**Background:**

Many hospitals in low- and middle-income countries cannot feasibly implement collection of two blood culture sets, which is required to meet the National Healthcare Safety Network (NHSN) coagulase-negative *Staphylococcus* (CoNS) BSI criteria. This study aimed to develop a predictive score for “true” BSI, using NHSN criteria as the gold standard, which may be useful in settings where only one set of blood cultures is available.

**Methods:**

We conducted a retrospective, cross-sectional study at a tertiary hospital. Adult inpatients (≥18 years) admitted from June 2020 to December 2022 with two or more blood culture (BC) sets collected within 24 hours and at least one set positive for CoNS were included. Exclusion criteria included hospital stay < 2 days, missing vital signs or endocarditis. Bivariate analyses compared CoNS BSI episodes to those that NHSN criteria were not fulfilled. Variables with a p ≤0.20 were selected through backward stepwise logistic regression; age, gender, number of BC sets collected, and COVID-19 infection were included regardless of p-value. Variable importance among selected predictors was assessed using XGBoost, then scaled and rounded into integer points without further transformation. Score performance was evaluated using ROC curve analysis; sensitivity and specificity were assessed across score thresholds.

**Results:**

We included 986 episodes (Figure 1). The patients characteristics and bivariate analysis are presented in Table 1. The predictors of CoNS BSI according to NHSN identified were shorter time to positivity (< 20 hours), presence of hypotension, *S. epidermidis* isolation, and neutropenia, resulting in an area under the curve (AUC) of 0.813. Variable importances were converted into score points (5, 3, 2, and 1, respectively), retaining a reasonable performance (AUC 0.717 - Figure 2). The sensitivity, specificity and accuracy of each score threshold is described in Table 2.

**Conclusion:**

A predictive score for “true” BSI was developed with reasonable accuracy. These variables formed the basis of a simplified point-based score intended for practical use in resource-limited settings. A cutoff ≥4 prioritized sensitivity, while ≥6 prioritized specificity. Threshold selection can align with surveillance or clinical priorities.

**Disclosures:**

All Authors: No reported disclosures

